# Changes in tobacco use at the early stage of the COVID-19 pandemic: Results of four cross-sectional surveys in Hong Kong

**DOI:** 10.18332/tid/145935

**Published:** 2022-03-04

**Authors:** Yuying Sun, Man Ping Wang, Yee Tak Derek Cheung, Sai Yin Ho, Tzu Tsun Luk, Shengzhi Zhao, Yongda Socrates Wu, Bonny Yee-Man Wong, Xue Weng, Jianjiu Chen, Xiaoyu Zhang, Lok Tung Leung, Kin Yeung Chak, Tai Hing Lam

**Affiliations:** 1 School of Public Health, The University of Hong Kong, Hong Kong SAR, China; 2 School of Nursing, The University of Hong Kong, Hong Kong SAR, China; 3 School of Nursing and Health Studies, Hong Kong Metropolitan University, Hong Kong SAR, China; 4 Institute of Advanced Studies in Humanities and Social Sciences, Beijing Normal University at Zhuhai, Zhuhai, China; 5 Department of Epidemiology, Mailman School of Public Health, Columbia University, New York, United States

**Keywords:** tobacco use, smoking, tobacco products, COVID-19, surveys and questionnaires

## Abstract

**INTRODUCTION:**

Changes in tobacco use since the COVID-19 outbreak differed by countries and little is known about changes in the use of specific tobacco products.

**METHODS:**

We analyzed data from four cross-sectional telephone/online surveys from April to June 2020 to investigate such changes since the 1st and 2nd wave outbreaks (February to April 2020) in Hong Kong. The respondents were 1595 adults (83.2% male) who used tobacco before the COVID-19 outbreak from our previous intervention study and surveys. We investigated the changes in tobacco use, intention to quit and quit attempts during the outbreak.

**RESULTS:**

About two-thirds (65.3%) of respondents reported no change in overall tobacco use, while 23.1% used less (including cessation) and 11.6% used more, resulting in a net decrease of 11.5 percentage points. A greater net decrease was observed for cigarettes (14.3% points) than heated tobacco products (HTPs, 3.3% points) and electronic cigarettes (e-cigarettes, 2.5% points). Decreased use was mainly due to the more extended stay at home (63.2%), health considerations (52.6%) and mask-wearing (47.4%), while increased use was for passing time (75.0%) and releasing stress (46.4%). Eight percent of cigarette, HTP and e-cigarette users reported a higher intention to quit since the pandemic outbreak. Nineteen percent of tobacco users (176/948) attempted to quit during the pandemic. Only 2.9% (46/1569) were abstinent at the time of the survey.

**CONCLUSIONS:**

Overall tobacco use decreased after the first two waves of the COVID-19 outbreak in Hong Kong. A greater proportion of cigarette users decreased use than HTP and e-cigarette users. Given the different methods used in the four surveys, future studies should aim for a large and representative sample.

## INTRODUCTION

Although the role smoking plays in the risk of contracting COVID-19 remains controversial^1-4^, smoking is likely associated with more severe outcomes of COVID-19^5,6^. Monitoring the impact of the pandemic on tobacco use behaviors is important for formulating future tobacco control policies. Some smokers have increased tobacco use during the pandemic to relieve mental distress^7^. The tobacco industry has opportunistically exploited the pandemic to promote alternative tobacco products^8^. Sales of heated tobacco products and e-cigarettes surged in the first quarter of 2020^9^. However, little is known about changes in the use of specific tobacco products. Some studies have examined the changes in tobacco use since the COVID-19 outbreak in the United States (US), United Kingdom (UK), Australia, Canada and other countries^10-20^. Some showed greater proportions of smokers who increased (19–45%) than decreased (13–28%) use^10-14^, while some reported greater decrease (15–73%) than increase (14–42%)^17-20^. Although among countries and regions with the lowest smoking prevalence, Hong Kong still has many daily smokers (691500; 10.2% of all persons aged >15 years)^21^. The changes in tobacco use in Hong Kong during the pandemic is unclear.

In Hong Kong, the 1st wave of the COVID-19 outbreak peaked on 9 February 2020 and the 2nd wave on 27 March 2020. To contain the outbreaks, the government implemented public health interventions (e.g. working from home) and restricted public gatherings to four people since 28 March 2020 but with no lockdown. Near universal voluntary masking in public places started in early February 2020. The first two waves were controlled with 1038 confirmed cases and four deaths by 27 April 2020. Face-mask wearing by the general public in Hong Kong was 97.5% in February and 98.8% in March 2020 from a telephone survey, and was 96.6% in April 2020 from an outdoor observational study^22,23^. The daily cases surged in early July, and the 3rd wave peaked on 30 July 2020 with 149 cases. In response, the government implemented mandatory indoor face-mask regulations on 23 July 2020^24^. The area has been expanded to outdoor public places since 29 July 2020. Starting from 28 August 2020, people who are doing strenuous outdoor exercise or having other reasonable excuses are exempted from wearing a mask, but smoking is expressly not exempted. The 4th wave that started in late November 2020 had decreased by April 2021.

There are few reports on the changes in heated tobacco product (HTP) and electronic cigarette (e-cigarette) use since the COVID-19 outbreak. Three surveys conducted in Belgium and the US on changes in e-cigarette use in adults showed higher proportions of decreased usage or quitting (15–40%) than increased use (10–25%)^20,25,26^. Two surveys showed a higher proportion of increased e-cigarette use (27%) than decreased use (16–24%)^27,28^ in the US. A survey reported higher proportions of HTP users increased use (31%) than decreased use or quitting (22%) in Japan^29^. Changes in the use of specific tobacco products in Hong Kong have not been reported. To provide more representative and comprehensive results, we analyzed data from four cross-sectional surveys conducted from April to June 2020 in Hong Kong to investigate changes in the use of different tobacco products in adults and their reasons since the first two waves of the COVID-19 outbreak (February to April 2020). The Supplementary file gives the STROBE Statement checklist of items that should be included in reports of observational studies.

## METHODS

### Respondents

The four cross-sectional surveys adopted different study designs and recruited respondents from several sources. All of the surveys were conducted online or by telephone. The respondents, aged ≥18 years, were all current tobacco users before the pandemic. The pre-pandemic period was defined as January 2020 or before, while the pandemic period referred to February to April 2020. All the surveys have been approved by the Institutional Review Board of the University of Hong Kong/Hospital Authority Hong Kong West Cluster (Surveys 1 and 3: UW20-238; Survey 2: UW20-326; Survey 4: UW19-169).

### Data collection

The results of two of our surveys (Surveys 1 and 3) have been published, focusing respectively on the misinformation that smoking can protect against COVID-19 and the perceived benefits and harms of COVID-19 on the family^30,31^. The present study includes Surveys 2 and 4 with data on specific tobacco products of cigarettes, HTPs, and e-cigarettes. Other products, including cigars, hand-rolled cigarette, water pipes and pipe smoking, were not included as such data were not collected. In addition, they accounted for only 0.5% of all tobacco products used in Hong Kong^21^.

Survey 1 was part of the COVID-19 Health Information Survey, a cross-sectional survey of Hong Kong adults aged ≥18 years, investigating COVID-19-related information use, preventive behaviors and well-being. The survey was conducted from 9 to 23 April 2020 through landline telephone and mobile phone surveys^30^. Briefly, respondents randomly sampled from the official numbering plan for landline telecommunication services were interviewed via telephone, while those from a mobile phone panel self-administered the survey online. Among the 2212 people invited, 1501 (67.9%) completed the survey, of whom 288 (19.2%) were current tobacco users before the pandemic.

Survey 2 was a telephone survey conducted from 7 May to 30 June 2020, including subjects who formerly participated in the ‘Quit to Win’ project for smokers in 2018 and 2019 (organized annually by the Hong Kong Council on Smoking and Health in collaboration with us). The ‘Quit to Win’ project has been reported elsewhere^32,33^. Among the 968 people invited, 769 (79.4%) agreed to participate. Those who had quit smoking before the COVID-19 pandemic were excluded. Current tobacco users before the pandemic were included in the present study (71.6%, 693/968).

Survey 3 was conducted from 26 to 31 May 2020 under the Jockey Club SMART Family-Link Project^31^. Both probability- and non-probability-based online panels through the Hong Kong Public Opinion Research Institute were invited. The probability-based panel included persons who were randomly selected in previous telephone surveys and representative of the Hong Kong population. In contrast, the non-probability-based panel included any person in Hong Kong who volunteered to join through completing an online form. An online questionnaire was sent to 70984 adults with valid email addresses. Of those who opened the invitation emails, 24.5% (4921 of 20103) provided useable data, and 4891 remained after excluding 30 respondents without family members. A total of 362 current tobacco users before the pandemic were included.

Survey 4 was conducted online from 3 to 29 June 2020 among current tobacco users recruited at outdoor smoking hotspots from July to November 2019 (response rate: 40.0%, 221/553). Three tobacco users were excluded as they had quit before the pandemic (roughly December 2019 to January 2020). A total of 218 respondents who were current tobacco users before the pandemic were included.

### Screening of current tobacco use

The definition of current tobacco use was in line with that of the HKSAR Government’s Thematic Household Survey^21^, but included alternative products such as HTPs and e-cigarettes.

Survey 1 was based on two questions: 1) ‘Are you currently using any tobacco products, e.g. cigarettes, HTPs, e-cigarettes? (Yes, daily; yes, non-daily; no, I have quit; no, I never used.)’; and 2) ‘Have you changed your smoking habits since the COVID-19 pandemic?’. Respondents were included if they: 1) were using tobacco daily/non-daily; or 2) had quit but answered ‘have slightly/considerably decreased use’ since the pandemic (quitters since the COVID-19 pandemic: quit all tobacco use at the time of survey but were a current tobacco user before the pandemic).

Survey 2 was based on two questions: 1) ‘Are you currently using any tobacco products, e.g. cigarettes, HTPs, e-cigarettes? (Yes, daily; yes, non-daily; no, I have quit)’; and 2) ‘How long have you quit?’ They were included if they: 1) were using tobacco daily/non-daily; or 2) quitters since the COVID-19 pandemic: had quit but less than three months (if the duration was over three months, i.e. before February 2020, the respondents were excluded as they had quit smoking before the pandemic).

Survey 3 was based on the same criteria as Survey 1.

In Survey 4, the eligibility was checked by a retrospective question: ‘In the past half-year (roughly from December 2019 to now), have you used any tobacco products?’ Affirmative respondents were included.

### Outcomes

The data on changes in tobacco use, intention to quit and quit attempts were extracted. Surveys 2 and 4 measured the changes in the use of cigarettes, HTPs and e-cigarettes separately. Survey 2 asked: ‘During the COVID-19 pandemic, have you changed the amount of tobacco consumption?’. Answers included ‘no change’, ‘increased’, and ‘decreased’. Survey 4 asked: 1) ‘What was the number of tobacco products you used per day before the pandemic?’; and 2) ‘What is the number of tobacco products you use per day now?’. The respondents answered the exact number of sticks for cigarettes and HTPs, and duration or number of puffs for e-cigarettes (10 minutes of use or 15 puffs were counted as one time of use). These were used to determine if tobacco consumption had increased, decreased, remained unchanged, or ceased (the numbers of all tobacco products were zero). Surveys 1 and 3 measured overall changes in tobacco use with the question: ‘Since the COVID-19 pandemic, have you changed your smoking habit?’. Answers were analyzed as increased, decreased and no change. For Surveys 2 and 4, respondents reported changes in the use of specific tobacco products. The overall changes were inferred under three scenarios: 1) If the respondent was using a single product, the overall change (increase or decrease) followed the single product; 2) If the respondent was using two or more products and all had changed in the same direction, the overall change was determined accordingly; and 3) If the respondent was using two or more products with changes in a different direction, overall change was coded as missing. The difference between the proportions of respondents who increased and decreased tobacco use was calculated as the net change in percentage points.

Surveys 2 and 4 measured the changes in tobacco use at home and outdoors by asking: 1) ‘Compared with pre-pandemic, have you changed your tobacco consumption at home since the pandemic?’. Answers included ‘decreased’, ‘no change’, ‘increased’, and ‘never used tobacco at home’; and 2) ‘Compared with pre-pandemic, have you changed your tobacco consumption outdoor since the pandemic?’. Answers included ‘decreased’, ‘no change’, ‘increased’, and ‘never used tobacco outdoor’. These two surveys also measured quit attempts during the pandemic by asking: ‘Since the pandemic, have you tried to quit smoking for a consecutive period of 24 hours?’. Answers included ‘yes’ and ‘no’. Survey 4 also included the reasons of increased or decreased tobacco use (answers were ‘mask-wearing’, ‘longer stay at home’, ‘health considerations’, ‘financial issues’, ‘no specific reasons’, ‘other reasons’ for decreased use, and ‘releasing stress’, ‘killing time’, ‘no specific reasons’, ‘other reasons’ for increased use), and changes in intention to quit (‘Compared with pre-pandemic, have you changed your intention to quit since the pandemic?’. Answers included ‘less intention to quit’, ‘no change’, ‘more intention to quit’ and ‘have quit’).

### Statistical analysis

The data from different surveys were merged to generate appropriate combined results, including 1561 current tobacco users (73.5% male). The analysis was conducted using SPSS 26.0 (IBM Corp, Armonk, NY). The number and percentage of categorical variables were reported. Sociodemographic characteristics were weighted by the sex and age distributions of current tobacco users of the 2019 Hong Kong adult population. However, we did not weigh sex and age by the type of tobacco products used due to a lack of data. Survey 1 adopted random sampling using landline telephone and mobile phone numbers. Survey 3 was based on a random sample from probability- and non-probability-based online panels, while Surveys 2 and 4 used convenient samples. As a sensitivity analysis, we also examined changes in overall tobacco use based only on Surveys 1 and 3 with random samples.

## RESULTS

Table 1 shows the characteristics of the four surveys. The original sample resulted in 1561 current tobacco users (73.5% male). The weighted sample included 1595 respondents (83.2% male) (Surveys 1 to 4: 287, 726, 360 and 222 respondents, respectively). The difference in sample size of the weighted and unweighted datasets was due to rounding. Surveys 1, 2 and 4 included 1202 respondents (aged 18–29 years: 8.1%; 30–39 years: 18.9%; 40–49 years: 21.8%; 50–59 years: 25.2%; and ≥60 years: 26.0%). Survey 3 included 360 respondents (aged 18–24 years: 2.8%; 25–34 years: 13.3%; 35–44 years: 20.6%; 45–54 years: 23.3%; 55–64 years: 23.1%; and ≥65 years: 16.9%). Age was missing in 35 respondents.

**Table 1:** Characteristics of the four studies, April–June 2020, Hong Kong

*Characteristics*	*Survey 1*	*Survey 2*	*Survey 3*	*Survey 4*
*Survey period*	*9–23 April n (%)*	*7 May–30 June n (%)*	*26–31 May n (%)*	*3–29 June n (%)*
**Sampling source/setting**	Landline telephone and mobile phone numbers	Actively approached in the community from all 18 districts in Hong Kong panels in 2018 and 2019	Probability- and non-probability-based online panels	Actively approached at smoking hotspots on streets during July to November 2019
**Survey mode**	Telephone & Online	Telephone	Online	Online
**Invited respondents**, n	2212	968	70984	553
**Response rate**	1501 (67.9)	769 (79.4)	4921 (6.9)	221 (40.0)
**Eligible current users**, n	288	693	362	218
**Unweighted sex** (% of male)	212 (73.6)	604 (87.2)	214 (59.1)	140 (64.2)
**Unweighted age** (years), range: mean (SD)	18–29: 44 (15.3) 30–39: 51 (17.7) 40–49: 55 (19.1) 50–59: 54 (18.8) ≥60: 84 (29.2)	18–29: 88 (13.6) 30–39: 158 (24.4) 40–49: 161 (24.8) 50–59: 119 (18.4) ≥60: 96 (14.8) Refused: 26 (4.0)	18–24: 6 (1.4) 25–34: 68 (15.6) 35–44: 150 (34.5) 45–54: 151 (34.7) 55–64: 55 (12.6) ≥65: 5 (1.1)	18–29: 55 (24.9) 30–39: 92 (41.6) 40–49: 51 (23.1) 50–59: 21 (9.5) ≥60: 2 (0.9)
**Weighted sample**	287	726	360	222
**Weighted sex** (% of male)	237 (82.6)	606 (83.5)	299 (83.1)	185 (83.3)
**Weighted age** (years), range: mean (SD)	18–29: 24 (8.4) 30–39: 54 (18.8) 40–49: 66 (23.0) 50–59: 71 (24.7) ≥60: 72 (25.1)	18–29: 54 (7.4) 30–39: 129 (17.8) 40–49: 148 (20.4) 50–59: 177 (24.4) ≥60: 184 (25.3) Refused: 35 (4.8)	18–24: 10 (2.8) 25–34: 48 (13.3) 35–44: 74 (20.6) 45–54: 84 (23.3) 55–64: 83 (23.1) ≥65: 61 (16.9)	18–29: 19 (8.6) 30–39: 44 (19.8) 40–49: 48 (21.6) 50–59: 55 (24.8) ≥60: 57 (25.7)
**Type of tobacco using**	Unknown	Cigarettes: 664 HTP: 134 e-cigarettes: 138	Unknown	Cigarettes: 203 HTP: 49 e-cigarettes: 25
**Outcomes**				
Changes in quantity of tobacco use	Overall	Cigarettes, HTP and	Overall e-cigarettes	Cigarettes, HTP and e-cigarettes
Changes of tobacco use at home and outdoors	N	Y	N	Y
Reasons of change	N	N	N	Y
Changes in quitting intention	N	N	N	Y
Quitting attempts	N	Y	N	Y

N: not reported. Y: reported. HTP: heated tobacco product. E-cigarette: electronic cigarette.

The results below were based on the weighted sample. Table 2 shows 65.3% of respondents reported no change in overall tobacco use, while 23.1% decreased use (including 2.9% who quit) and 11.6% increased use, resulting in a net decrease of 11.5 percentage points. If the respondents of only Survey 1 and Survey 3 were included, the net decline in overall tobacco use was 16.2 percentage points. Twenty-two percent cigarette users decreased use while only 8.1% increased use, resulting in a net decrease of 14.3 percentage points. The vast majority of users of HTP (92.3%) and e-cigarette (91.4%) reported no change in consumption, resulting in smaller net decreases of 3.3 and 2.5 percentage points, respectively.

**Table 2:** Changes in tobacco use during the COVID-19 pandemic, April–June 2020, Hong Kong

*Change in use*	*Tobacco use*			
	*Cigarettes (Surveys 2 & 4) n (%)*	*HTPs (Surveys 2 & 4) n (%)*	*E-cigarettes (Surveys 2 & 4) n (%)*	*Any tobacco product (Surveys 1– 4) n (%)*
**Unweighted sample**				
No change	545 (66.2)	173 (88.3)	152 (87.4)	950 (62.1)
Increased	74 (9.0)	7 (3.6)	9 (5.2)	190 (12.4)
Decreased	204 (24.8)	16 (8.2)	13 (7.5)	344 (22.5)
Quit*	-	-	-	46 (3.0)
Total	823	196	174	1530
Net change, n (decreased percentage points)	130 (15.8)	9 (4.6)	4 (2.3)	200 (13.1)
**Weighted sample^†^**				
No change	603 (69.6)	169 (92.3)	149 (91.4)	1024 (65.3)
Increased	70 (8.1)	4 (2.2)	5 (3.1)	182 (11.6)
Decreased	194 (22.4)	10 (5.5)	9 (5.5)	317 (20.2)
Quit *	-	-	-	46 (2.9)
Total	867	183	163	1569
Net change, n (decreased percentage points)	124 (14.3)	6 (3.3)	4 (2.5)	181 (11.5)

* For specific tobacco products, data were only available in Survey 4 and unavailable in Surveys 1–3, because the general question ‘Are you currently using tobacco products, e.g. conventional cigarettes, heated tobacco products, electronic cigarettes (at the time of survey)?’ could not identify from which tobacco products the respondents had quit (those who selected ‘No, I quit smoking’).

† The proportions were weighted by sex and age in current tobacco users of 2019 Hong Kong adult population.

Survey 4 showed that among the tobacco users who decreased use, the reduced amount were 4.5 sticks (SD=1.72; n=19) of cigarettes, 5.2 sticks (SD=3.52; n=8) of HTPs and 3.4 times (SD=3.68; n=3) of e-cigarette use. Among the tobacco users who increased use, the increase was 5.7 sticks (SD=3.15; n=28) of cigarettes, 4.0 sticks (SD=1.83; n=3) of HTPs and 3.4 times (SD=5.88; n=3) of e-cigarette use.

In Survey 4, the main reasons for decreased cigarette use (n=19) were longer stay at home (63.2%), health considerations (52.6%), mask-wearing (47.4%) or financial issues (15.8%). Increased cigarette use (n=28) was most commonly for killing time (75.0%), releasing stress (46.4%), no specific reasons (21.4%) or other reasons (14.3%). According to two dual users of HTPs and cigarettes who chose other reasons, HTPs were difficult to obtain during the pandemic. Survey 4 showed most users had no change in intention to quit (86.7% cigarette users, 87.8% HTP users, and 72.0% e-cigarette users). In comparison, 8.4% cigarette users, 8.1% HTP users, and 8% e-cigarette users reported more intention to quit or have quit during the pandemic compared with pre-pandemic.

The pooled data of Surveys 2 and 4 showed that 18.6% of tobacco users (176/948) had attempted to quit (abstained from all tobacco products for at least 24 hours) during the pandemic (Survey 2: 20.5%; Survey 4: 12.2%). Among 176 respondents (Surveys 2 and 4) who attempted to quit, 30 (17.0%) were abstinent at the time of the survey. The combined data from four surveys showed only 2.9% (46/1569) were abstinent at the time of the survey. The pooled data of Surveys 2 and 4 showed that 51.9% decreased use outdoors (Survey 2: 60% decreased, increased data not available; Survey 4: 27.5% decreased vs 3.6% increased), while 22.1% increased use at home (Survey 2: 25.2% increased, decreased data not available; Survey 4: 12.6% increased vs 5.0% decreased). Survey 4 showed 64.1% of respondents had no changes in tobacco use both at home and outdoors, 16.4% had no changes at home but decreased use outdoors, 7.7% increased use at home but decreased use outdoors, and 3.6% increased use at home but had no changes outdoors.

## DISCUSSION

We gathered the data of current smokers from four cross-sectional surveys, which were based on different sampling frames. Data from individual surveys were included for specific analyses only if the surveys had collected such data. A greater proportion of adult tobacco users reported a decrease (23.1%, including quitting) than an increase (11.6%) in tobacco use since the first two waves of the COVID-19 pandemic, resulting in a net decrease of 11.5 percentage points. Including only the respondents from the random samples resulted in a net reduction of 16.2 percentage points. However, it is possible that the changes are natural fluctuations and may not be attributed to the pandemic. During the first two waves in Hong Kong, 18.6% of tobacco users had attempted to quit. But of those who tried to quit, only 17% were abstinent at the time of survey. One of our surveys (Survey 4) showed that the main reasons for increasing consumption were for passing time or releasing stress. Some people might have become unemployed and taken no-pay leave or worked from home since the pandemic, resulting in stress and boredom. Our previous article reported that more days stayed-at-home were associated with more depressive symptoms since the COVID-19 epidemic^34^. Our results indicate the importance of providing smoking cessation services or assistance, using online approaches when face-to-face sessions are not allowed by social distancing regulations, together with stress coping strategies, particularly for those who had quit intention or attempts.

We observed minor changes in HTP and e-cigarette use since the COVID-19 pandemic. Some previous studies showed that e-cigarettes and HTPs helped smokers quit cigarettes, but many smokers would keep using e-cigarettes or HTPs. A Cochrane review has found a higher smoking cessation rate using nicotine e-cigarettes than nicotine replacement therapy^35^, but the outcome was not abstinence from all tobacco products. A trial in England found 80% of participants who quit cigarettes using e-cigarettes were still using them at 12 months^36^. A cohort study showed e-cigarette use could not help abstinence from all tobacco products^37^. A study in Italy has also shown that during the COVID-19 pandemic, most exclusive cigarette smokers had considered quitting, but most exclusive e-cigarette users had not^15^. A UK study found that current dual-use of cigarettes and e-cigarette was associated with 2.15-fold higher odds for reporting COVID-19 infection^38^. Many HTP and e-cigarette dual users of cigarettes were more nicotine dependent than exclusive cigarette users^39,40^. Our previous prospective study in Hong Kong showed HTP use was not associated with cigarette abstinence at six months^41^. In the present study, 9 in 10 HTP and e-cigarette users reported no changes in use during the COVID-19 pandemic, and only two had reduced use due to difficulties in purchasing HTPs. HTPs have not been launched in Hong Kong, but some users acquired HTPs through travelling to other countries before restrictions were implemented due to the pandemic or through online purchase. The Hong Kong SAR Government has passed a new law that bans the import, manufacture, sale, distribution and advertisement of alternative smoking products (e-cigarettes, HTPs, herbal cigarettes) on 21 October 2021 with a grace period of 6 months. Monitoring and intervening against tobacco use, including HTPs and e-cigarettes, must be continued before and after enforcement of the law, especially if the pandemic remains.

We found that 22% of respondents had increased tobacco use at home, which would increase secondhand smoke exposure of family members. Our previous study showed longer stay at home was significantly associated with increased tobacco use^30^. As COVID-19 is likely to have major impacts for several years globally, compulsory mask-wearing in public places with no exemption for outdoor smoking has been implemented in Hong Kong since 29 July 2020. Monitoring the trends of tobacco consumption, indoor smoking prevalence and quitting, concerning various pandemic control measures during the pandemic are warranted.

### Limitations

Our study had some limitations. The four surveys adopted different study designs and recruited respondents from various sources. The changes in tobacco use were self-reported, and data on reduced use could be subject to social desirability bias. Furthermore, the reasons for changes in tobacco use and intention to quit were only based on one survey. This would be better to be explored by a qualitative study. Lastly, the samples might not be representative of all current smokers. For example, Survey 4 recruited more young tobacco users. Surveys 3 and 4 included more female users. The response rate in Survey 3 was lower than the other surveys probably because email invitations did not involve any direct contact (voice or face-to-face) with the respondents. Although we weighted the data by sex and age using population-based data to reduce bias, the heterogeneity among surveys was a major limitation of the present study.

## CONCLUSIONS

We found a net decrease of 11.5 percentage points in tobacco use amidst the early phase of the COVID-19 pandemic. More cigarette users decreased use than HTP and e-cigarette users. Given the different methods used in the four surveys, future studies should aim for a large and representative sample. Qualitative studies are warranted to explore the mechanisms of behavioral changes to support future smoking cessation research and services.

## Data Availability

The data supporting this research are available from the authors on reasonable request.
